# Optimizing Soil Stabilization with Chitosan: Investigating Acid Concentration, Temperature, and Long-Term Strength

**DOI:** 10.3390/polym17020151

**Published:** 2025-01-09

**Authors:** Runshen Wang, Dominic E. L. Ong, Hossein Sadighi, Mohammad Goli, Peng Xia, Hadi Fatehi, Tianchi Yao

**Affiliations:** 1 Key Laboratory of Geomechanics and Embankment Engineering of Ministry of Education, Hohai University, Nanjing 210024, China; panda.jasonwang@gmail.com (R.W.); xiapeng_1994@163.com (P.X.); 2 Cities Research Institute, Griffith University, Southport, QLD 4215, Australia; 3 School of Engineering and Built Environment, Griffith University, Nathan, QLD 4111, Australia; 4 Department of Civil Engineering, Isfahan University of Technology, Isfahan 84156-83111, Iran; h.sadighi@alumni.iut.ac.ir; 5 Department of Civil, Ocean, Environmental Engineering, Stevens Institute of Technology, Hoboken, NJ 07030, USA; mgoli1@stevens.edu; 6 College of Science, Australia National University, Canberra, ACT 2600, Australia; u7886714@anu.edu.au

**Keywords:** biopolymer, chitosan, environmentally friendly, cost-effective

## Abstract

Civil and geotechnical researchers are searching for economical alternatives to replace traditional soil stabilizers such as cement, which have negative impacts on the environment. Chitosan biopolymer has shown its capacity to efficiently minimize soil erosion, reduce hydraulic conductivity, and adsorb heavy metals in soil that is contaminated. This research used unconfined compression strength (UCS) to investigate the impact of chitosan content, long-term strength assessment, acid concentration, and temperature on the improvement of soil strength. Static triaxial testing was employed to evaluate the shear strength of the treated soil. Overall, the goal was to identify the optimum values for the mentioned variables so that the highest potential for chitosan-treated soil can be obtained and applied in future research as well as large-scale applications in geotechnical engineering. The UCS results show that chitosan increased soil strength over time and at high temperatures. Depending on the soil type, a curing temperature between 45 to 65 °C can be considered optimal. Chitosan biopolymer is not soluble in water, and an acid solution is needed to dissolve the biopolymer. Different ranges of acid solution were investigated to find the appropriate amount. The strength of the treated soil increased when the acid concentration reached its optimal level, which is 0.5–1%. A detailed chemical model was developed to express how acid concentration and temperature affect the properties of the biopolymer-treated soil. The SEM examination findings demonstrate that chitosan efficiently covered the soil particles and filled the void spaces. The soil was strengthened by the formation of hydrogen bonds and electrostatic interactions with the soil particles.

## 1. Introduction

The rapid growth of the world’s population has increased the socio-economic demand for the development and expansion of civil infrastructure at an unprecedented rate. The construction of civil foundations requires proper soil improvement methods; more than 40,000 soil improvement projects with a total cost of more than 6 billion dollars per year are implemented worldwide [1]. The main purpose of ground improvement is to enhance the bearing capacity, shear strength, soil stiffness, resistance improvement against surface erosion, and soil hydraulic conductivity and seepage [2,3,4,5,6]. Climate change and global warming are two of the most concerning trends of the last several years. Regarding the materials used in construction projects, both synthetic and conventional materials with a calcium basis, including cement and lime, have contributed to the production of greenhouse gases that are directly linked to global warming. The production of one unit of cement emits one ton of carbon dioxide, accounting for five to eight percent of global carbon dioxide emissions [7,8,9,10].

Soil stabilization methods are usually divided into three main categories: mechanical, biological, and chemical. Conventional chemical stabilizers such as cement and lime are still the most common materials in large-scale soil improvement projects. However, their excessive use leads to significant environmental issues, including CO_2_ emissions during cement production, suppression of plant growth, groundwater pollution, and global warming. In contrast, biological approaches such as microbially induced calcite precipitation (MICP), enzyme-induced calcite precipitation (EICP), and biopolymer application in soil enhancement have attracted greater attention in the field of geotechnical engineering due to their minimal negative effects on the environment [11,12,13,14].

Biopolymers are natural polymeric products that are environmentally friendly and sustainable, with high cohesive properties, and can be widely found in the environment in different forms [15,16,17,18]. These properties make them a suitable choice for soil improvement applications. Biopolymers, using natural polymers such as polysaccharides, have several advantages for engineering purposes in the enhancement of soil structure, water retention, and the reduction in wind, water, and unwanted vegetation growth erosion [19,20]. However, the widespread use of biopolymers is not still very common in engineering fields due to their relatively high costs. The costs of biopolymer production and application have significantly reduced over the past years. Polysaccharides are known for their high potential and effectiveness in improving soil properties [21,22,23,24].

Chitosan is a natural polysaccharide derived from chitin as well as the exoskeletons of crustaceans such as crabs and shrimps [25]. Chitosan has soil-binding, water-retention, and biodegradable properties. Several studies have been conducted to examine the potential of chitosan in soil enhancement [26]. It was shown that adding chitosan improved the mechanical properties of different types of soils (clay and sand) by positively increasing the interparticle interaction of the soil particles and biopolymer. Chitosan was presented as an ecologically acceptable substitute for conventional additives, such as cement and lime, when Jamshidi et al. (2023) investigated its impact on marl soil qualities and found that the polymer greatly enhanced soil shear wave velocity and maximum shear modulus by 51 and 128% after 7 days curing even after 8F-T cycles compared to those of unstabilized samples [27]. Ilman et al. (2023) examined the impact of chitosan on earthen construction materials. Chitosan was shown to act as a reliable stabilizer, enhancing the mechanical properties of the soil and preventing the development of visible cracks. This is explained by the creation of a stable framework that connects the particles of the soil, therefore increasing its durability [28]. Additionally, it was found by Adamczuk et al. (2022) that chitosan behaves differently in different types of soils [29]. No secondary environmental impacts have been observed when using chitosan as an adhesive in soil improvement applications, aligning with the goal of transitioning from traditional to new-generation materials in geotechnical engineering [30].

The number of studies on the use of chitosan as a soil stabilizer is limited; however, there is still a significant gap in exploring all aspects of this research field. This paper aims to investigate the behavior of chitosan-treated soil under various curing temperatures, long-term curing periods, and different acidity concentrations of chitosan solution. Biopolymer content and soil particle size are additional parameters examined in this research. These parameters were evaluated using the unconfined compressive strength (UCS) test. Consolidated drained (CD) static triaxial testing was employed to examine how optimal conditions of these parameters influence the shear strength of kaolinite and sand. SEM images were included to provide visual insights into chitosan-treated sand and kaolinite. Additionally, chemical analysis was conducted to assess how temperature and the acidity concentration of the biopolymer solution affected soil properties. Upon completing this study, we aim to deepen understanding of chitosan-treated soil and the effectiveness of different parameters in geotechnical engineering applications.

## 2. Materials

### 2.1. Kaolinite

The kaolinite silt used in this research was purchased from Kaolin Malaysia, a mining products specialist. The kaolinite was taken from depths of 4.0 to 6.0 m below the ground surface. Table 1 shows the results of the XRD analysis performed on the kaolinite silt. The composition of kaolinite is 78% silt, 21.12% clay, and 0.88% sand, as determined by geotechnical criteria derived from particle size distribution and hydrometer tests (Table 2). According to the Unified Soil Classification System (USCS), it is classified as high-plasticity kaolinite silt (MH) with a Skempton activity rating of 0.77%. This description illustrates its versatility, necessitating a thorough examination of its properties and behavior in order to optimize its utilization.

### 2.2. Sand

**Figure 1:**
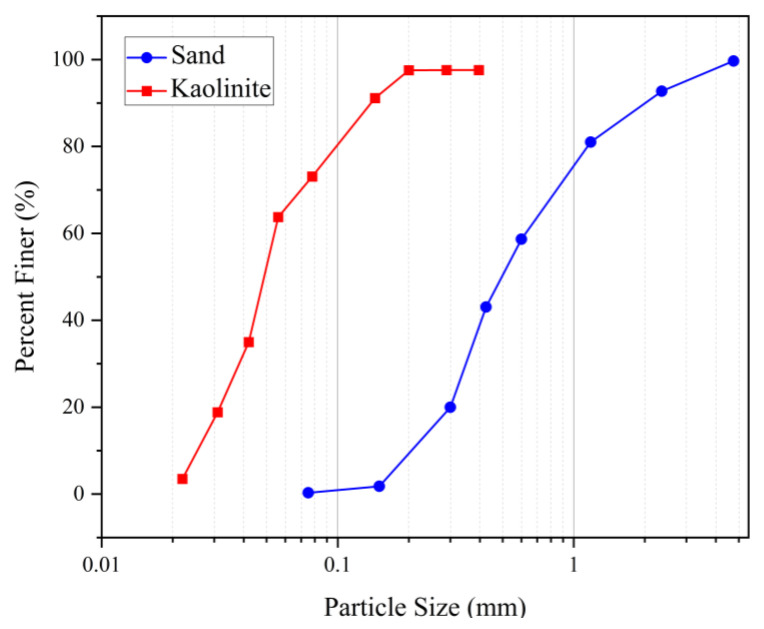
Particle size distribution of kaolinite silt and sand.

The sand employed in this study was obtained from a construction site located in Gold Coast, Australia. According to the USCS classification, it is classified as poorly graded sand (SP). The gradation analysis of the utilized kaolinite silt and sand is depicted in Figure 1. The values of the gradation coefficient (Cc) and uniformity coefficient (Cu) are 2.77 and 0.91, respectively. The void ratio of the sand varies between 0.61 and 0.76. The XRD analysis of the sand’s chemical composition is detailed in Table 3, whereas the geotechnical properties are presented in Table 4.

### 2.3. Chitosan

**Figure 2:**
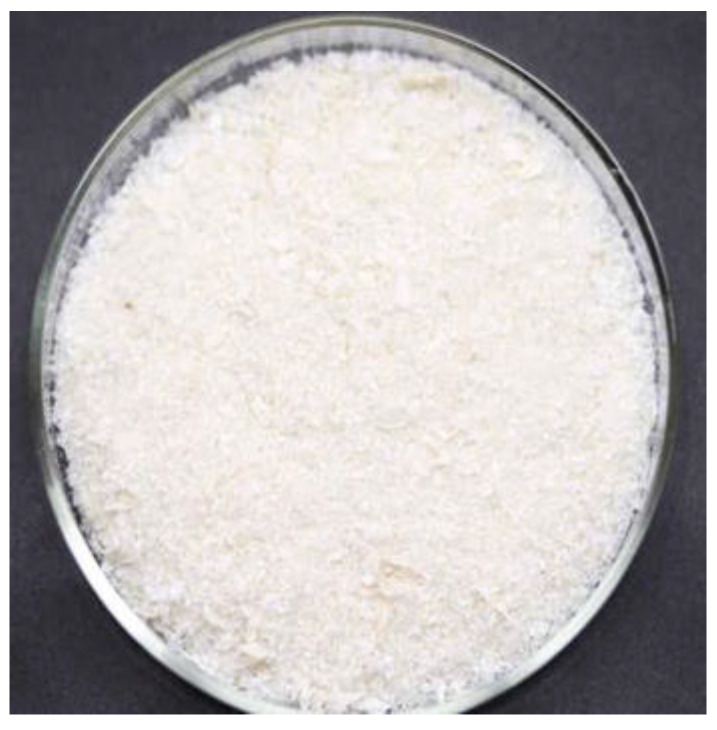
Chitosan biopolymer used in this study.

For Chitosan manufacturing objectives, the United States, Japan, India, Canada, China, South Korea, Russia, and Norway frequently employ the byproducts of crustacean fisheries. The principal industrial source from which the biopolymer is extracted is fishery industry detritus, specifically the shells of prawns, crabs, and lobsters. An estimated 100 billion kilograms of chitin and its derivatives are produced on an annual basis. Because of its strong hydrogen bonds and polymeric structure, chitin is insoluble in most organic acids and water. With CAS No. 9012-76-4, the Chemsupply firm provided the chitosan that was used. Table 5 and Figure 2 provide specifics on the chitosan biopolymer. The molecular weight (MW) of the chitosan was determined to be 120–140 kDa, and the degree of acetylation (DA) was approximately 15%.

**Figure 3:**
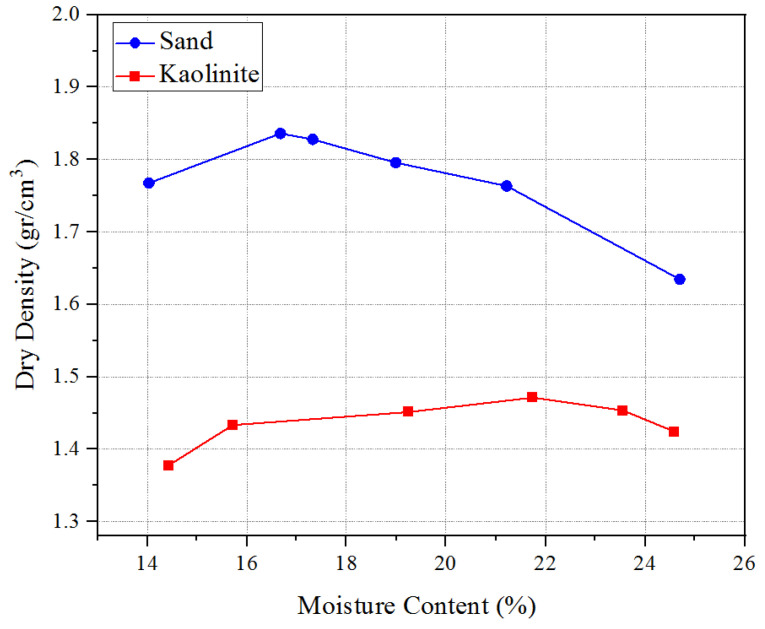
Compaction curves for sand–kaolinite mixtures.

A standard compaction test was conducted to determine the maximum dry density and optimum moisture content of the soil. This information was important for calculating the appropriate amounts of soil and water needed to prepare the samples for both untreated and treated soils. The experiment was performed based on the ASTM D698 [31] standard and the results are shown in Figure 3.

## 3. Research Method

### 3.1. Sample Preparation

To attain maximum efficiency, the wet mixing method was implemented, in which, first, the biopolymer dissolves in the solvent until reaching a desirable concentration and then the biopolymer solution is mixed with soil. To prepare the biopolymer solution, the following steps were taken: (a) the solution was heated to 80 °C after dissolving the specified amount of glacial acetic acid in distilled water to obtain a 1% concentration; (b) chitosan powder was added to the acetic acid solution as required; and (c) constant stirring continued until a homogeneous solution was obtained, ensuring that the concentration remained moderate enough to dissolve all components.

The soil was subsequently mixed with the solution for 10 min after being dehydrated in an oven set at 105 °C for 24 h. The mixture was then packed in a bag for a day to ensure even moisture distribution. A small sample was taken and placed in the oven to determine its moisture content.

A specially designed mold was fabricated in compliance with ASTM D2850 [32] and ASTM D4609 [33] guidelines to ensure consistency in the sample preparation. A cylindrical stainless-steel tube with two wings attached to the sides served as the mold’s main component. A square stainless-steel plate, a plug, short and long hammers, and other supporting components were included. The cylinder’s fixed dimensions were 50 mm in diameter and 200 mm in height. During the sample fabrication process, lubrication was provided to the inside walls of the tube, the plug, and the short hammer’s surfaces. The square plate made it easier to modify the mold’s location once the mixture was put inside. Using a hydraulic jack, compaction was carried out in a single layer until the maximum density determined by compaction tests was exceeded by 90%. Following compaction, the samples were extruded and cured in a controlled setting at a temperature of 25 °C and a relative humidity of 50–60%. The cylindrical samples that were selected for triaxial and UCS testing had dimensions of 100 mm in height and 50 mm in diameter.

### 3.2. Experimental Program

This study involved various experiments, including compaction, UCS, static triaxial testing, and SEM imaging, to assess the impact of the biopolymer on the engineering properties of the soil. UCS and triaxial tests were used to evaluate the mechanical properties of the biopolymer-treated soil, and SEM photography was used to provide a microstructural representation of the biopolymer–soil interaction. Table 6 shows the experimental procedures for this study.

Details of the different parameters investigated in this study can be found in Table 6. Biopolymer content, long-term curing assessment, acid concentration, and temperature were evaluated through the UCS test. Samples were kept for up to 120 days to see how the compressive strength of the biopolymer-treated soil changed over time. Acid concentrations are a crucial factor in preparing chitosan solutions; therefore, different concentrations were prepared to evaluate this parameter. For the temperature effect, samples were cured from 15 to 80 °C.

### 3.3. Unconfined Compression Test

A universal testing machine was used to evaluate the compressive strength in accordance with ASTM D2166 [34]. UCS experiments were utilized to determine the ideal values for the following factors: temperature, soil type impact, acid concentration, biopolymer content, and long-term strength assessment. The device monitored and recorded stress–strain behavior as the axial strain rate increased from 1%/min (1 mm/min) to 7% of the total strain. The average of the three specimens evaluated in each condition was used to calculate the unconfined compressive strength.

### 3.4. Infiltration Test

**Figure 4:**
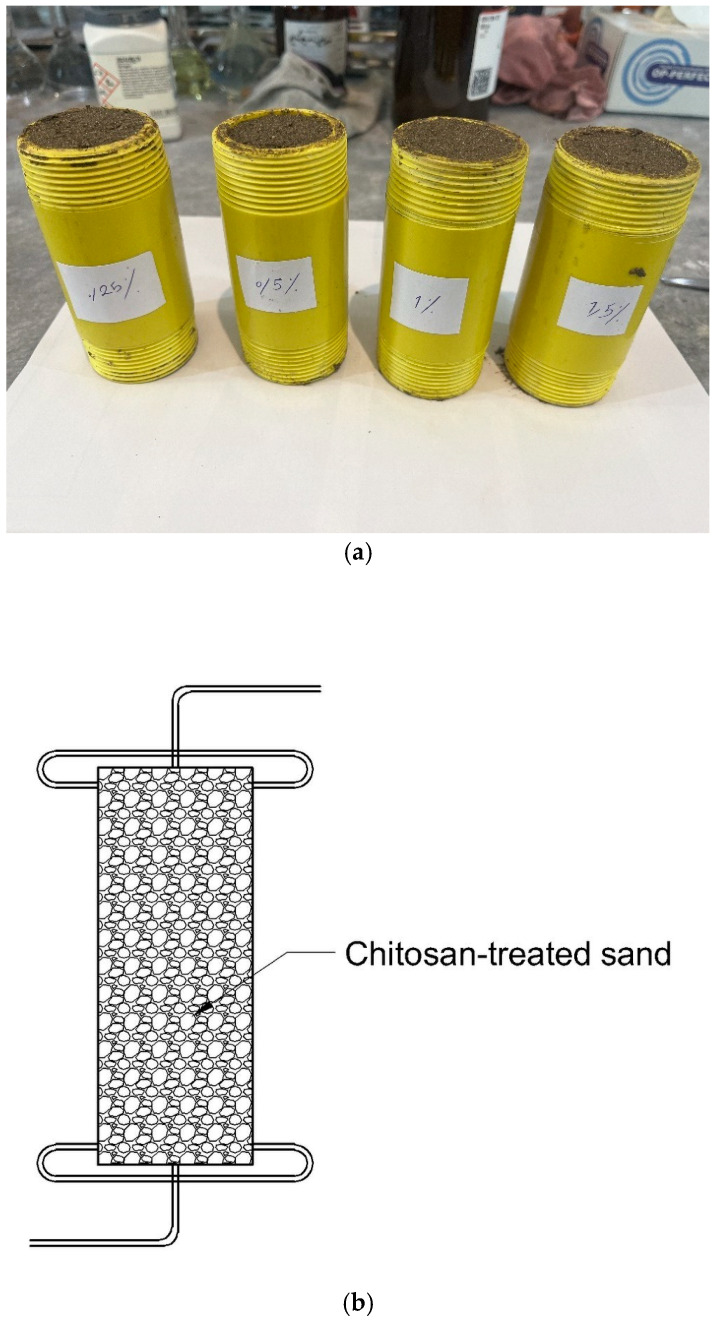
(**a**) Infiltration samples and (**b**) schematic form of the mold.

To study the impact of water flow on the UCS of chitosan-treated soil, a controlled infiltration test was designed and performed. The test included samples placed inside a polyethylene cylinder with inner diameter of 50 mm and height of 100 mm. Each sample was enclosed with a cap tightened at both the top and bottom. A 10 mm diameter hole was made in the center of each cap, into which a small tube was inserted to facilitate water entry and exit. The overall shape of the sample can be seen in Figure 4.

Water entered the samples through the upper cap, and a constant hydraulic head of 2 m was maintained to ensure a steady flow through the sample. Water exited through the lower cap, and the water flow was sustained for 8 h. Following the infiltration process, the samples were carefully extracted from the tubes and allowed to air dry for 14 days. After the drying period, UCS tests were performed to assess the extent of biopolymer loss due to water flow and its subsequent effect on the compressive strength of the treated samples.

### 3.5. Static Triaxial Test

The triaxial specimens were prepared under the same circumstances used for the UCS test, and they were then cured in a controlled atmosphere at 25 °C and around 60% relative humidity. Consolidated undrained (CU) triaxial testing was carried out in accordance with ASTM D7181 specifications [35].

**Figure 5:**
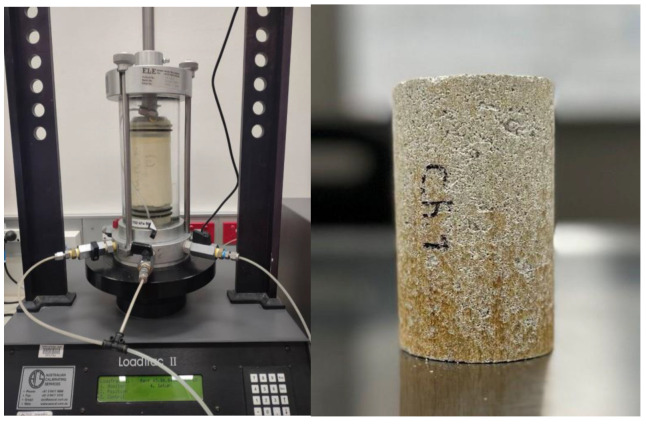
Triaxial testing system.

In order to evaluate the impact of chitosan on sand and kaolinite, a series of static triaxial experiments were implemented. The initial confining pressure of each sample was kept at 100 kPa. Static triaxial testing was carried out on both saturated and dry samples. In order to facilitate the breakdown of air within the pore space into deaired water, saturation was achieved by maintaining an effective stress of 20 kPa while applying back pressure from the sample’s bottom. Saturation persisted until a B value greater than 95% was obtained, indicating successful saturation. A small amount of consolidation was expected at this step due to the sample preparation using a hydraulic jack, so a quick consolidation under 50 kPa pressure was applied as a safety precaution. The applied strain rate was fixed at 0.1 mm/min during the shearing step. The overall shape of the system used for the triaxial testing is shown in Figure 5.

### 3.6. Scanning Electron Microscopy (SEM) Imaging

SEM images were obtained to investigate the microscopic interactions and structural characteristics of the biopolymer and soil. The motivation behind using SEM was to observe the detailed surface morphology and bonding between chitosan and the soil particles. For SEM characterization, soils treated with biopolymers as well as untreated soil (sand, kaolinite) were used. To ensure dryness, the samples were dried for 24 h at 25 °C in the oven. Carbon-conductive tabs were then used to attach them to a SEM mount. After applying a coating of carbon paint to guarantee adequate electric grounding, conductive tape was used to cover the samples. For specimen observation, the Zeiss Sigma VP Field Emission Scanning Electron Microscope (Oberkochen, Germany) was utilized.

## 4. Results

### 4.1. Unconfined Compression Strength (UCS) Test

#### 4.1.1. Biopolymer Content Effect on the Compressive Strength of Chitosan-Treated Soil

**Figure 6:**
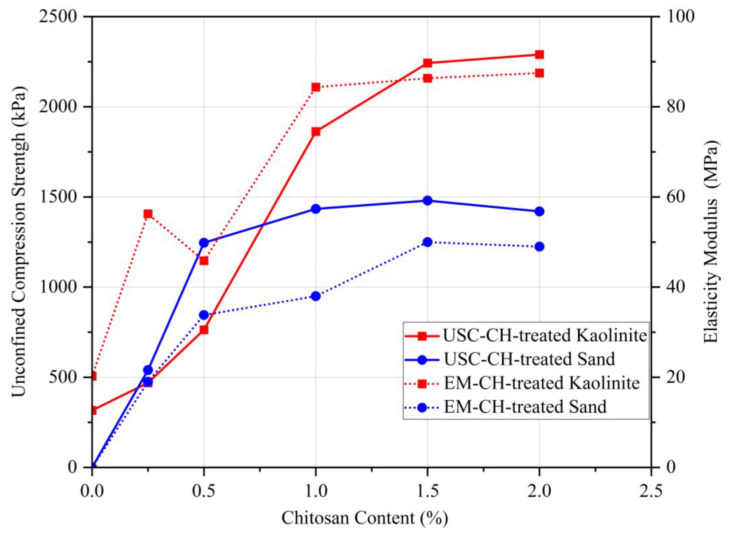
Effect of chitosan content on the mechanical behavior of CH-treated soils’ compressive strength and elasticity modulus.

Unconfined compression strength (UCS) testing plays an important role in evaluating biopolymer influence in soil treatment by measuring the maximum compressive load that a sample can withstand. Figure 6 shows the UCS and modulus of elasticity (EM) values for biopolymer-treated sand and kaolinite soils with varying amounts of chitosan (0, 0.25, 0.5, 1, 1.5, and 2%) at a curing temperature of 25 °C for a period of 14 days. From the compaction test, the optimal moisture content for this test for sand and kaolinite soil was determined as 17 and 35%, respectively. Figure 6 shows that EM represents the elasticity modulus, and UCS stands for the unconfined compressive strength.

According to Figure 6, chitosan biopolymer increased the UCS of soils regardless of the soil type. The compressive strength of untreated sand and kaolinite was obtained as 0 and 316 kPa, respectively. Adding 0.25% chitosan increased the compressive strength to 540 kPa for the treated sand and 469 kPa for the treated kaolinite, indicating that sand outperformed kaolinite in the chitosan-treated soil. Up to 0.5% chitosan, sand still maintained a higher strength. The coarse-grained nature of sand facilitated more effective mixing with chitosan compared to kaolinite, which was primarily responsible for this improvement.

Kaolinite’s compressive strength exceeded that of sand after adding 1% chitosan, highlighting its superior bonding and integration with the finer particles of kaolinite. The optimal compressive strength for stabilized sand was obtained as 1480 kPa with 1.5% chitosan, while for stabilized kaolinite, it was 2290 kPa with 2% chitosan. In conclusion, the addition of chitosan to sand and kaolinite soils forms bonds that enhance soil strength [16]. However, the UCS test indicates that, with increasing chitosan content, kaolinite soil exhibits greater strength compared to sand soil. This is due to various bonding mechanisms: (a) hydrogen bonding between chitosan and hydroxyl kaolinite surface; (b) electrostatic interaction between charged surfaces of kaolinite and chitosan; (c) hydrophobic bonding; and (d) covering kaolinite particles with chitosan chains to create a strong and integrated network in the particles. It prevents soil particles from shifting and increases soil strength. Sand particles’ lack of significant electric charge makes electrostatic and hydrogen bonding phenomena unlikely.

Figure 6 shows that the addition of chitosan increases the soil’s modulus of elasticity, which is similar to compressive strength. The modulus of elasticity of untreated sand and kaolinite was obtained as 0 and 20 MPa, respectively. Sandy soil lacks cohesion between its grains and therefore cannot withstand any significant strength. As a result, its modulus of elasticity was obtained as zero. On the other hand, kaolinite soil has a cohesive nature between its grains, which allows it to withstand some strength. This cohesive characteristic gives the kaolinite soil a non-zero modulus of elasticity. However, when 1.5% chitosan is added, the modulus of elasticity slightly decreases for the treated sand but stays constant for the treated kaolinite. Due to its lubricating effects, adding more chitosan to the soil results in a decrease in soil particle cohesion. Additionally, by distributing polymeric chains of biopolymers across the soil void space and covering the sand surfaces, a robust and well-integrated film network is created throughout the treated soil mass, improving the resistance forces and preventing soil grain movement. The optimal modulus of elasticity with 2% chitosan sand was 49 MPa, while for kaolinite, it was 86 MPa with 1.5% chitosan.

#### 4.1.2. Long-Term Curing Time Effect on the Strength Behavior

The impact of chitosan treatment over time is investigated in this section. Samples were kept up to 120 days so that the effect of long-term curing could be studied using a UCS test. The samples were prepared at a temperature of 25 °C using a biopolymer solution with an acidity concentration of 10 g/L and a chitosan content of 0.5%. Various curing times were considered for the samples, including 0, 1, 3, 7, 14, 28, 60, 90, and 120 days.

**Figure 7:**
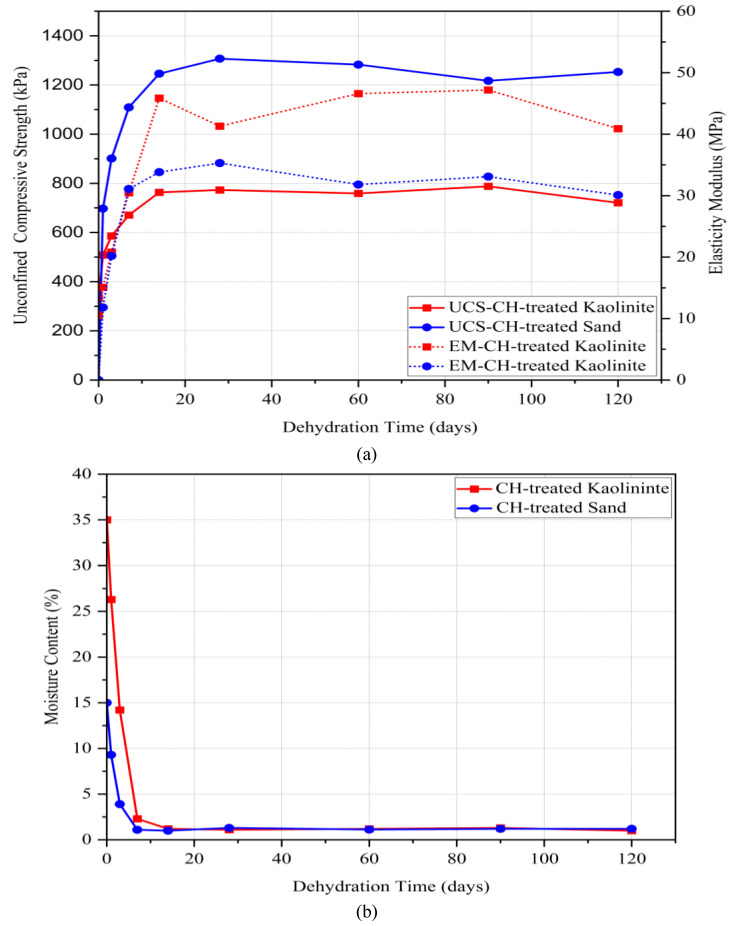
Effect of long-term dehydration on the UCS of CH-treated soils. (**a**) Compressive strength and elasticity modulus, (**b**) moisture content.

Figure 7a shows the UCS and modulus of elasticity as well as the moisture content over time for sand and kaolinite soils treated with chitosan over time. As curing time progressed, the UCS of both treated soils increased regardless of the soil type. At the time right after sample preparation, the UCS of the treated kaolinite was around 334 kPa, while the treated sand showed zero strength due to the lack of cohesion between the soil grains. After one day of dehydration, the strength of the treated sand increased dramatically to 697 kPa, which was higher than that of the treated kaolinite. The reason is that sand has a more porous structure, making moisture loss easier compared to the treated kaolinite with much finer particles.

After seven days, approximately 90% of the UCS was achieved for both treated soils, indicating that the size of soil particles does not significantly impact the results after one week. Moisture content was reduced to less than 3%, as curing time has an inverse relationship with moisture content. Samples reached their final strength at 14 days after preparation, which was around 760 kPa for the treated kaolinite and 1250 for the treated sand. No significant changes in compressive strength were observed from 14 days to 120 days, demonstrating that the bonds between the biopolymer and soil particles maintained their integrity over four months. Additionally, the moisture content remained below 1.5%, indicating that the samples were almost completely dried, with only small amounts of moisture retained from the ambient humidity. The decline in humidity over a period of 14 days was a significant contributing factor to the rise in UCS. Moisture is crucial for dissolving chitosan in the solvent (a water and acetic acid solution). As the water content decreases over time, the formed gel becomes stronger and more brittle. This process enhances the polymeric chains of chitosan, creating a stronger soil–biopolymer mass, which results in increased compressive strength.

#### 4.1.3. Temperature Effect on Compressive Strength of Biopolymer-Treated Soil

**Figure 8:**
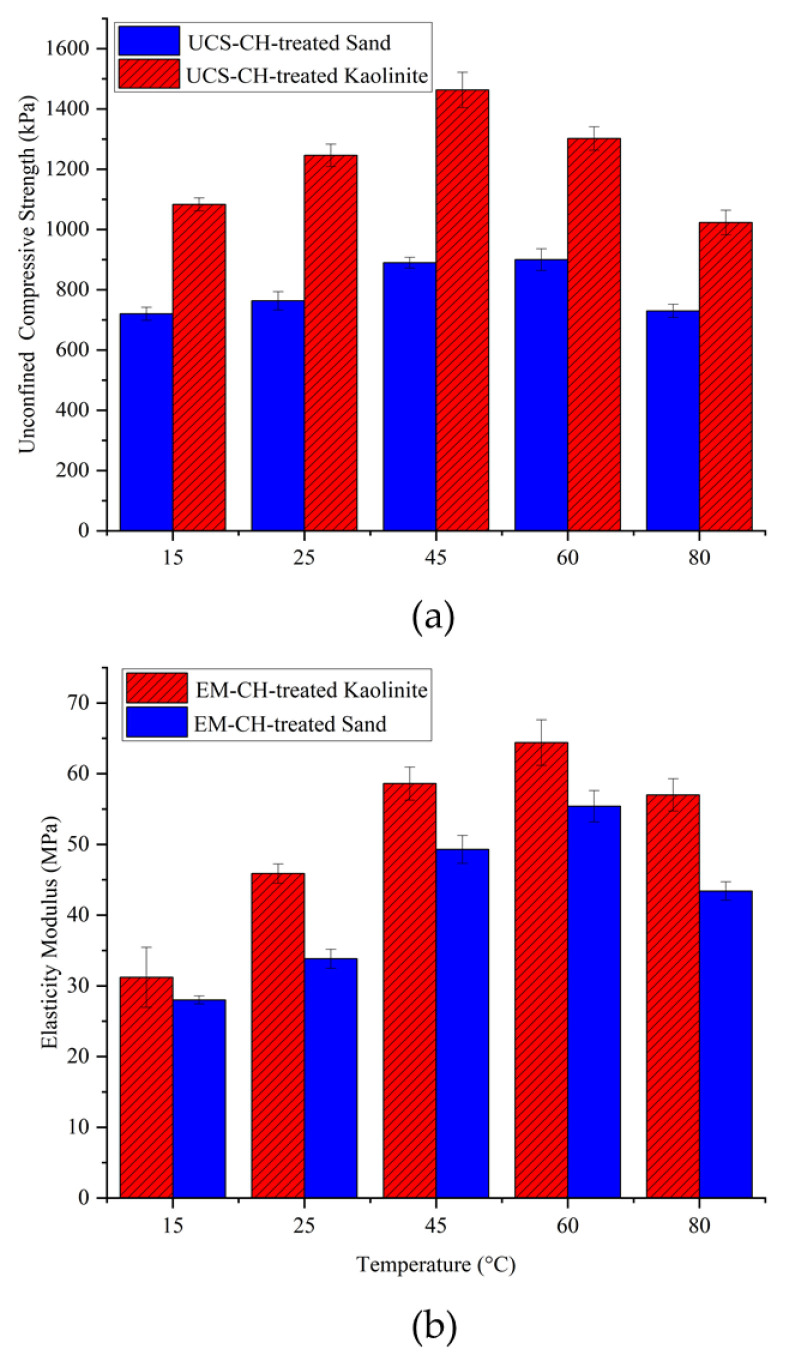
Effect of temperature on the UCS of CH-treated soils. (**a**) Compressive strength, (**b**) elasticity modulus.

Due to the biodegradability of biopolymers, environmental temperatures play a crucial role, especially at higher temperatures. To examine the effect of temperature on biopolymer-stabilized kaolinite and sand, samples were cured at temperatures of 15, 25, 45, 60, and 80 °C and then tested using UCS tests. The samples were kept at the specified temperatures for 14 days, with a chitosan content of 0.5% and an acidity concentration of biopolymer solutions of 10 gr/L. Figure 8 shows the UCS for the biotreated sand and kaolinite soils versus temperature. As shown, the compressive strength of both treated soils increased with the elevation of temperature up to 45 °C. For the chitosan-treated kaolinite, 60 °C was the peak UCS, after which it started to decrease at 80 °C. For the chitosan-treated sand, the UCS decreased after 45 °C, indicating its optimal dehydration temperature. The increased soil compressive strength from 15 to 45 °C was due to the dehydration of biopolymer gel and forming stronger chemical bonds.

From the elasticity modulus graph, it can be observed that very high temperatures (80 °C) led to a reduction in the stiffness of the samples. The reason is that the chitosan hydrogel shrinks due to dehydration, forming brittle chitosan fibers and involving a smaller surface area of soil particles in the bonding mechanisms. This effect is especially pronounced in treated sand, which has no electrical charge on its particle surfaces, resulting in smaller adhesion surface contact between the biopolymer film and soil grains and consequently lower compressive strength parameters. On the other hand, biopolymers have the ability to produce more gel at higher temperatures; therefore, increasing the temperature causes chitosan to generate more gel, which leads to soil improvement.

**Figure 9:**
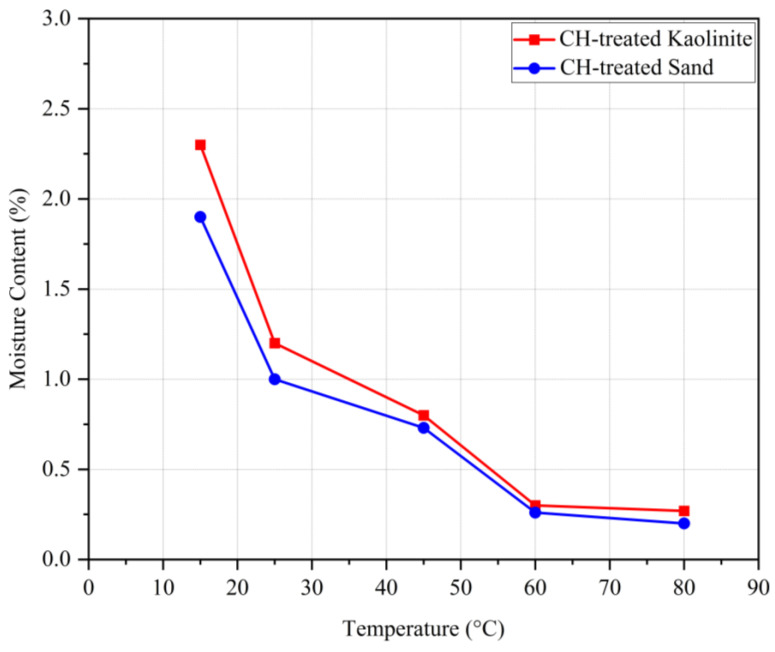
Effect of temperature on the UCS of CH-treated soils.

Figure 9 shows the variation of moisture content for samples cured at different temperatures. As seen, higher temperatures resulted in lower moisture content after 14 days of dehydration. An inverse correlation is observed between moisture content and UCS. Referring to the obtained results, it can be said that very low (around 15 °C) and very high (above 60 °C) temperatures result in relatively lower compressive strengths compared to the samples cured within this range. This is because lower temperatures retain higher moisture content, resulting in more flexible biopolymer films, whereas higher temperatures absorb almost all the moisture, leading to brittle biopolymer films.

#### 4.1.4. Acid Concentration Effect on the Behavior of the Treated Soil

Chitosan is a biopolymer that is insoluble in neutral water with a pH around 7. To prepare a chitosan solution, the pH needs to be lowered, as chitosan is soluble in diluted acid solutions. Acetic acid is commonly used for this purpose. The dissolution of chitosan in a diluted acid involves the protonation of its amine groups (-NH_2_), making it soluble. The chemical reaction for dissolving chitosan in a diluted acid is as follows:

Chitosan-NH_2_ + CH_3_COOH → Chitosan-NH_3_^+^ + CH_3_COO^−^

Chitosan needs a sufficient degree of protonation of its amino groups (-NH2) to dissolve completely in the acid solution. At lower concentrations, there may not be enough acetic acid molecules available to effectively protonate the chitosan molecules. Conversely, higher concentrations of acetic acid may overly protonate the chitosan molecules. An excessive positive charge can alter the structure and properties of chitosan and affect its solubility characteristics. Very high concentrations of acid can also lead to the precipitation of chitosan due to the strong acidic environment. Therefore, investigating how acid concentrations affect the behavior of chitosan-treated soil is of high importance. For this purpose, various concentrations of acid were prepared, including 0, 0.1, 0.2, 0.3, 0.4, 0.5, 1, 2, and 5%, with the biopolymer content set at 0.5% of the soil weight. Similar to the previous sections, kaolinite and sand were the soils used in this study. The treated samples were dehydrated for 14 days at a temperature of 25 °C.

**Figure 10:**
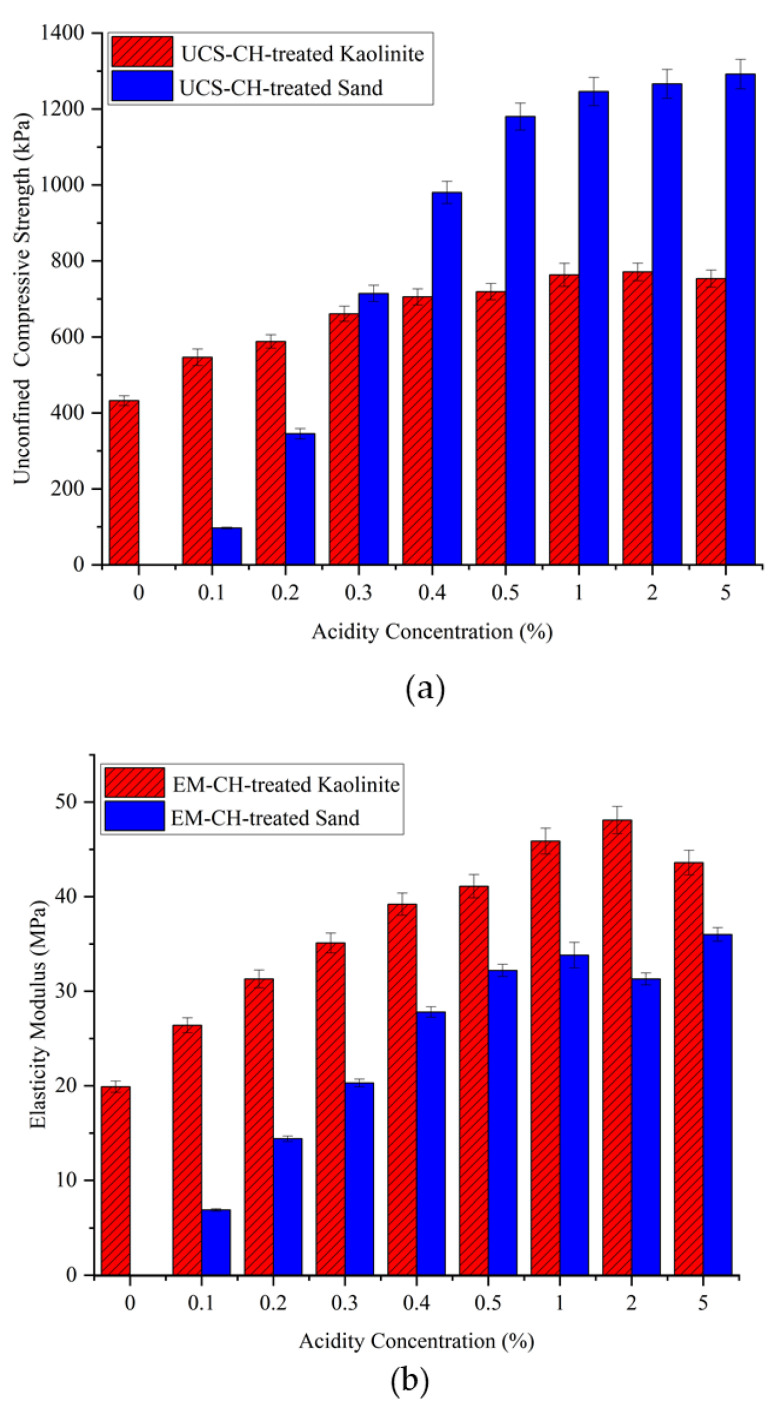

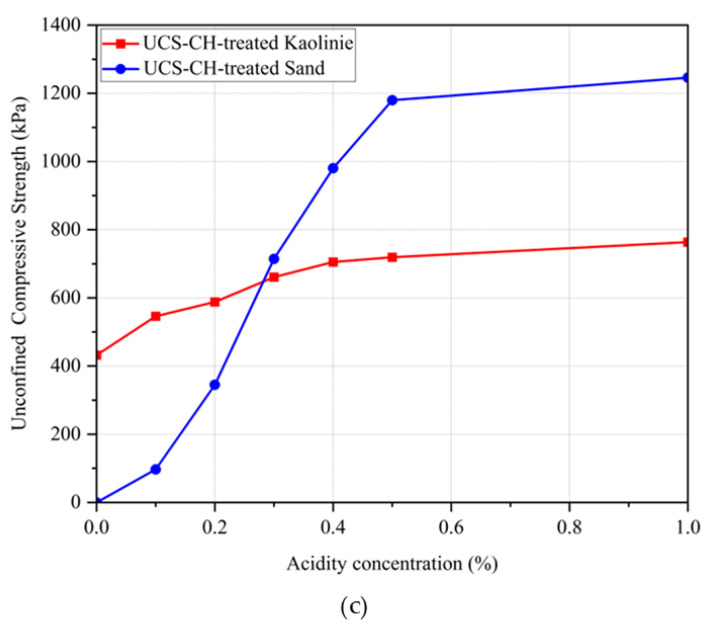
Effect of acid concentration on the UCS of CH-treated soils. (**a**) Compressive strength, (**b**) elasticity modulus, (**c**) variation of UCS vs. acidity concentration between 0–1%.

The mechanical properties of the chitosan-treated soil can be seen in Figure 10. At 0% acid concentration, chitosan is approximately insoluble in neutral water, resulting in chitosan precipitating in the solution with only a small amount dissolving. Consequently, the sand samples were not able to maintain their structure, yielding zero strength. For the chitosan-treated kaolinite, only a slight improvement in the UCS was observed. Adding a small amount of acid to water (0.1%) significantly increased the compressive strength from 432 kPa to 546 kPa for the treated kaolinite and from zero to around 100 kPa for the treated sand. This increasing trend remained very promising up to a 0.5% acidity solution, indicating that a large percentage of chitosan dissolved into the solution. According to the literature, 0.5% to 1% acidity solution is commonly used for most chitosan applications, suggesting that this range provides enough acid molecules to effectively dissolve chitosan. However, at a 5% acid concentration, no significant change in the UCS was observed.

**Figure 11:**
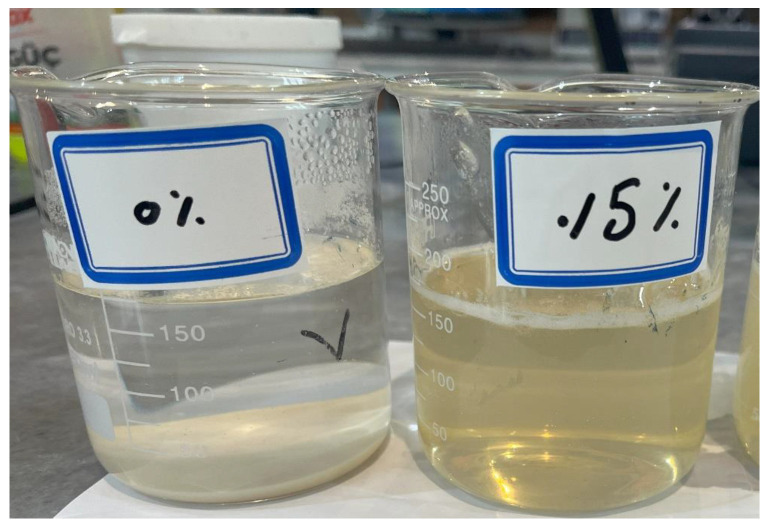
Chitosan in water vs. low concentration acid (0.5%): dissolution comparison.

In real-world applications, whether temporary or long-term, acid rain is a potential concern regarding the leaching of biopolymer from chitosan-treated soil. According to available data, the typical acid concentration in acid rain ranges from 0.00002 to 0.00006 g/L. These concentrations are significantly lower than the levels required to solubilize chitosan, which necessitates much higher acid concentrations (1000 to 50,000 mg/L or 0.1% to 5%). Figure 11 shows a comparison between the solutions with and without enough acid concentration.

### 4.2. Infiltration Test

**Figure 12:**
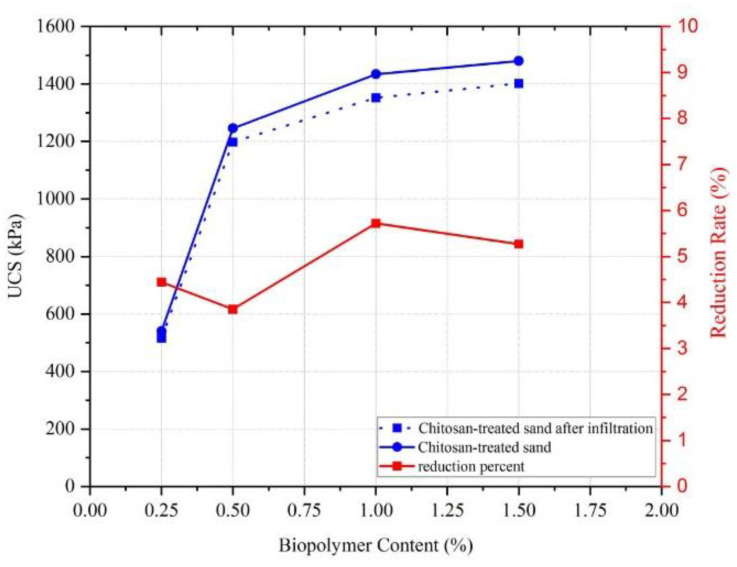
Compressive strength change for chitosan-treated sample after infiltration test.

Figure 12 shows the results obtained from the UCS tests before and after the infiltration process. The results prove that chitosan exhibits a remarkable resistance to a consistent water flow with minimal reduction in the compressive strength. For 0.25% chitosan, a decrease of 4.4% was observed in UCS, from 540 kPa to 516 kPa. Similarly, the 0.5% chitosan-treated sample saw a decrease from 1246 kPa to 1198 kPa, with a reduction percentage of just 3.85%. The higher chitosan content samples, 1% and 1.5%, experienced reductions in UCS from 1434 kPa to 1352 kPa and from 1480 kPa to 1402 kPa, corresponding to reductions of 5.72% and 5.27%, respectively.

These results clearly demonstrate that the infiltration process led to only slight decreases in compressive strength, which indicates that a small amount of the chitosan was washed away. This underscores the potential of chitosan as a reliable additive in civil and geotechnical applications, especially in environments where the soil may be subjected to water infiltration over time. Chitosan’s insolubility in water ensures that its chemical structure remains intact, providing sustained stability and effectiveness as a soil stabilizer even under prolonged water exposure.

### 4.3. Static Triaxial Test

A set of consolidated drained (CD) triaxial tests were conducted on the untreated and chitosan-treated kaolinite samples, as well as on untreated and chitosan-treated sand, to investigate how chitosan biopolymer affects the shear strength properties of different soil types. The sample preparation followed the same procedure as the UCS samples, with compression applied using a hydraulic jack to achieve a dry density higher than 95% of the maximum dry density (MDD). Chitosan was used at a concentration of 0.5% (mb/ms) to treat both kaolinite and sand samples. The samples were allowed to dehydrate for 14 days at a temperature of 25 °C.

**Figure 13:**
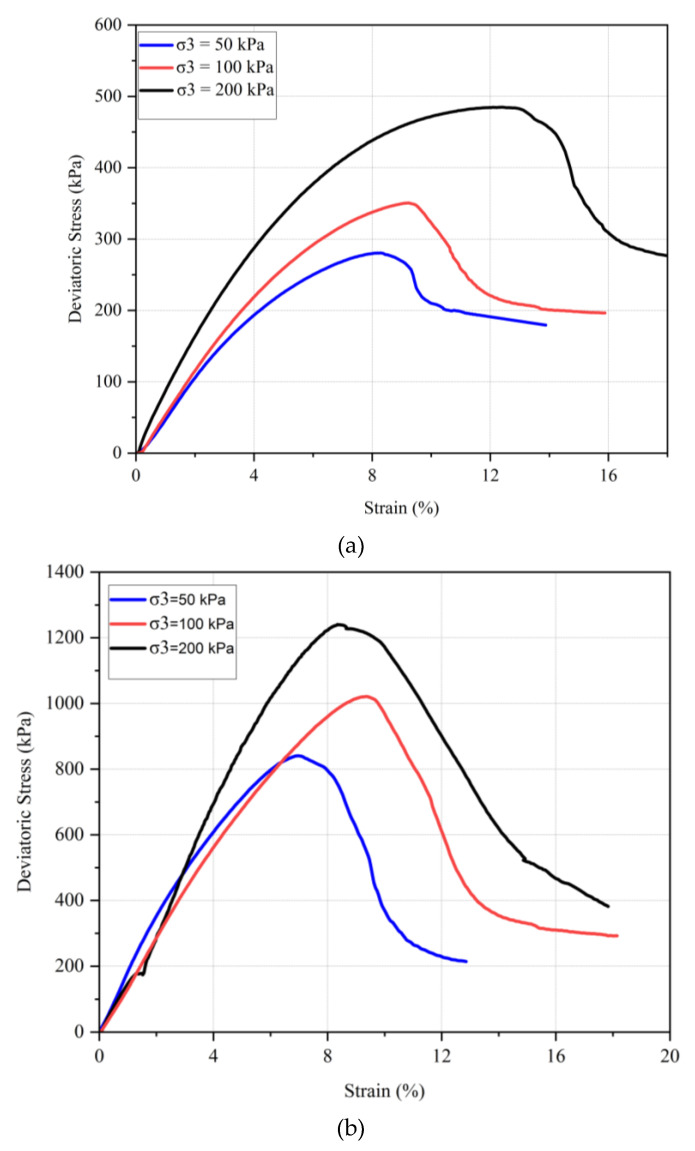
Stress–strain behavior of (**a**) untreated kaolinite and (**b**) chitosan-treated kaolinite.

**Figure 14:**
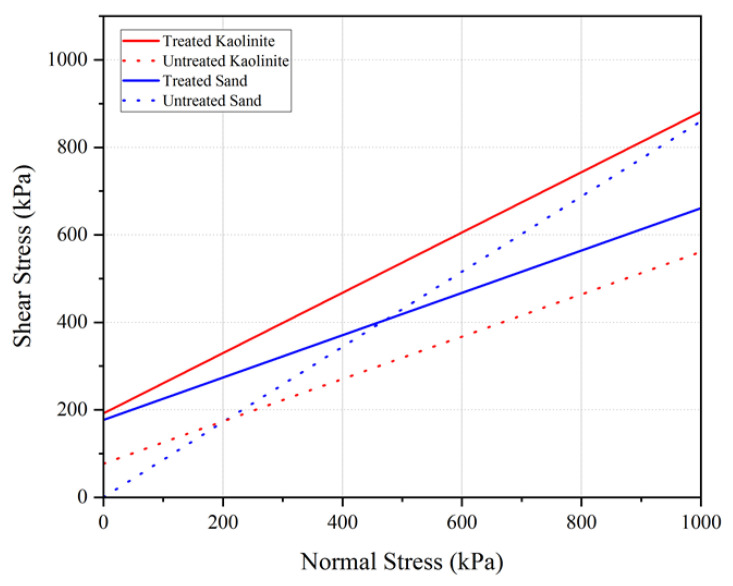
Failure envelope of treated and untreated kaolinite.

Three different confining pressures (50, 100, and 200 kPa) were applied consistently across all tests to compare the shear strength at failure for different samples. The results are illustrated in Figure 13 shows the stress–strain behavior of untreated and chitosan-treated kaolinite at confining pressures of 50, 100, and 200 kPa. The failure envelopes for the tested samples are presented in Figure 14.

The data indicate that for both untreated and chitosan-treated kaolinite, an increase in confining pressure corresponds to an increase in strength. For instance, the maximum deviatoric stress for untreated kaolinite increased from 280 kPa to 350 kPa and further to 490 kPa when the confining pressure was increased from 50 kPa to 100 kPa and 200 kPa, respectively. In contrast, the chitosan-treated kaolinite samples exhibited a significant enhancement in shear strength, with deviatoric stresses rising from 840 kPa to 1020 kPa and 1240 kPa under the same confining pressures. Residual strengths for the chitosan-treated samples were also notably higher compared to the untreated kaolinite.

Similarly, for the chitosan-treated sand, the deviatoric stresses were recorded as 520 kPa, 651 kPa, and 785 kPa under confining pressures of 50, 100, and 200 kPa, respectively. The chitosan-treated sand also demonstrated substantial improvements in shear strength parameters, as shown by the cohesion value of 161 kPa and a friction angle of 39.5°, compared to untreated sand, which had a cohesion of 0 kPa and a friction angle of 36°.

Table 7 summarizes the Mohr–Coulomb shear strength parameters for the untreated and chitosan-treated samples.

The results indicate a substantial improvement in the shear strength parameters for chitosan-treated kaolinite and sand. The effective cohesion (C’) of kaolinite increased from 70 kPa for untreated kaolinite to 192 kPa for chitosan-treated kaolinite, while the effective internal friction angle (φ’) increased from 23.76° to 34.58°. Similarly, for sand, the cohesion increased from 0 kPa to 161 kPa and the friction angle increased from 36.04° to 39.5° upon treatment with chitosan. This improvement can be attributed to the adhesive properties of chitosan, which enhances particle bonding and soil cohesion. The formation of chitosan–soil conglomerates during compaction further contributes to the increased interlocking and frictional resistance within the soil matrix.

In conclusion, the treatment of kaolinite and sand with 0.5% chitosan significantly enhances their shear strength, demonstrating the potential of chitosan as an effective soil stabilizer. The notable increase in both cohesion and friction angle underscores the efficacy of chitosan in improving soil mechanical properties, making it a viable option for various geotechnical applications.

### 4.4. Acid Concentration and Temperature Effects on the Interaction of Chitosan and Soil

**Figure 15:**
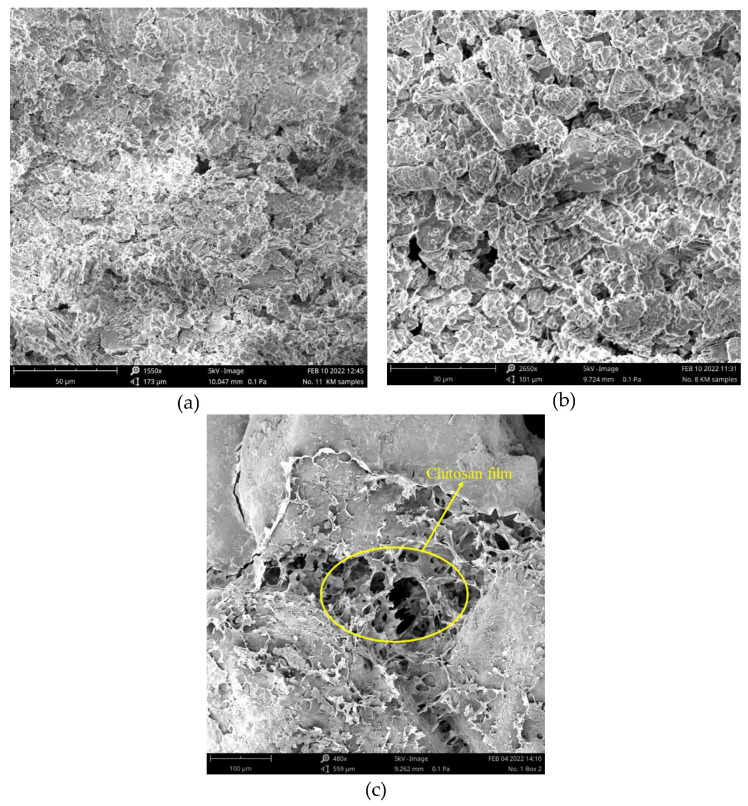
SEM images from untreated and treated samples. (**a**) Untreated kaolinite, (**b**) chitosan-treated kaolinite, (**c**) chitosan-treated sand.

Kaolinite, a hydrous aluminum phyllosilicate, is one of the most abundant clay minerals in Earth’s crust and belongs to the dioctahedral 1:1 kaolin mineral group. In its structure, oxygen atoms bonded to silicon atoms form a tetrahedral sheet, referred to as the siloxane surface. Similarly, hydroxyl groups bonded to aluminum atoms form an octahedral alumina sheet, known as the aluminol surface. As depicted in Figure 15a, both sheets share the apical oxygen atoms. Higher MW polysaccharides may exhibit increased tensile strength but reduced solubility, while lower MW variants may lack sufficient bonding capabilities. Each kaolinite layer is considered as a strong dipole, where the aluminol surface is hydrophilic and dominated by positive charges, while the siloxane surface exhibits negative charges and is hydrophobic. Thus, the kaolinite layers are held together by strong hydrogen and dipolar interactions [36]. The edges of these layers consist of O atoms and OH groups. In an acidic solution (pH < 3.6), the hydroxyl groups accept protons from the solution, forming protonated OH groups. Conversely, at pH > 3.6, they release protons into the solution, resulting in O⁻ ions. Therefore, the kaolinite surfaces exhibit two types of charges: a permanent negative charge on the tetrahedral face and a variable pH-dependent charge, either positive or negative, due to the protonation or deprotonation of hydroxyl groups at the amphoteric sites, including the edges and the octahedral faces [37,38]. Figure 15 shows scanning electron microscopic (SEM) images of untreated and treated soils.

**Figure 16:**
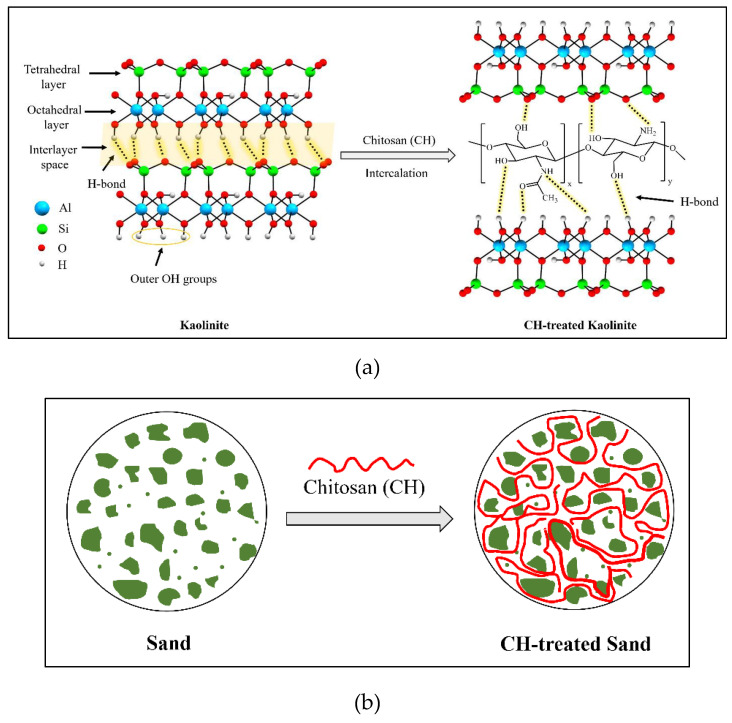
Interaction of chitosan biopolymer with soil. (**a**) Chitosan-treated kaolinite, (**b**) chitosan-treated sand.

The molecular structure of chitosan is also shown in Figure 16a. Chitosan, the deacetylated form of chitin, is one of the most common polymers found in nature and is composed of N-acetylated glucosamine and glucosamine units [39]. The degree of deacetylation (DD) determines the content of free amino groups (NH2) in the polysaccharides, which can be used to differentiate between chitin and chitosan [40]. Chitosan is a weak base that exhibits low solubility in organic solvents and is completely insoluble in neutral and alkaline environments. However, it dissolves in dilute aqueous acidic solutions (pH < 6.5), which can convert glucosamine units into the soluble form R-NH_3_^+^ according to the following chemical reaction:

R-NH_2_ + AcOH ↔ R-NH_3_^+^ + AcO^−^

where R-NH_2_ represents the free amine form of chitosan, while AcOH denotes the undissociated form of acetic acid. R-NH_3_^+^ represents the protonated form of chitosan, which is soluble in water, and AcO^−^ is the acetate counter ion [41].

The functional groups in both structures play an important role in the formation of the kaolinite–chitosan composite. Chitosan contains several active functional groups, including amine (-NH2), hydroxyl (-OH), and ketone (-C=O). These groups can form hydrogen bonds with the hydroxyl groups present on the surface of kaolinite (Figure 16a). This interaction improves the adhesion between chitosan and kaolinite in the composite. In acidic conditions (pH < 6.5), another potential interaction occurs between the NH_3_+ groups of chitosan and the negatively charged surface of kaolinite, involving electrostatic forces [42,43].

The major component of river sand is the SiO_2_ compound. Unlike kaolinite, the sand does not have enough active and available functional groups to establish significant hydrogen bonding with chitosan functional groups. Two primary driving forces can be identified in studying the interactions between sand and chitosan in the composite: (1) interfacial mechanical interaction and (2) hydrophobic bonding. The interfacial mechanical interaction enhances the composite structure by creating polymer bonds around the sand grains, as illustrated in Figure 16b. In addition, a hydrophobic interaction occurs at the interface between the polymer fibers containing nonpolar carbon chains and the sand grains with uncharged surfaces [6].

**Figure 17:**
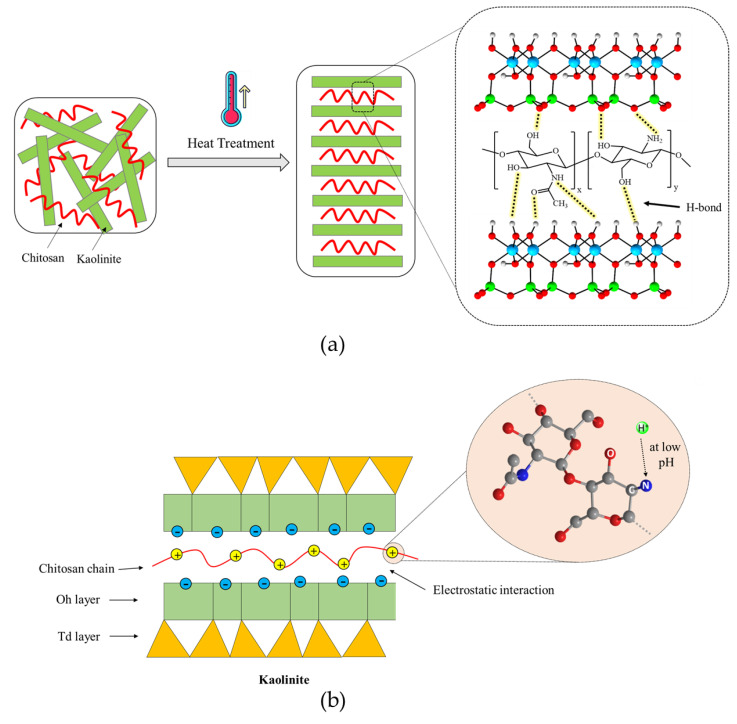
Effects of various parameters on the properties of chitosan-treated soil. (**a**) Temperature, (**b**) Acid concentration.

The acid concentration and temperature are important factors that may affect the interactions in the composites. Heat treatment is important for modifying the microstructure and properties of chitosan composites. The temperature most probably modifies hydrogen bond distribution and improves chitosan–clay interactions. As shown in Figure 17a, increasing the temperature breaks down the hydrogen bonds that connect polymer chains. This encourages a more open structure, which enables cross-linking interactions. In addition, heat treatment reduces the moisture content and leads to the compaction of the composite structure. It should be noted that there is a temperature threshold known as the ceiling temperature. This temperature is critical because it marks the point above which the efficiency of the reaction or the strength of cross-linking interactions begins to decrease. Additionally, higher temperatures can enhance the mobility of chitosan chains, facilitating better interaction and bonding with soil particles. This can lead to increased tensile strength and durability of the composite. However, if the temperature exceeds the optimal range, it can cause thermal degradation of chitosan, weakening the composite structure. Proper temperature control is essential to maintaining the balance between enhanced interaction and preventing thermal damage. Furthermore, temperature variations can influence the rate of moisture evaporation, which plays a crucial role in the final stabilization of the soil composite [44,45,46].

Figure 17b illustrates the mechanism demonstrating the necessity of sufficient acid for dissolving chitosan. The adsorption of chitosan onto kaolinite is mainly driven by electrostatic attraction between positively charged chitosan molecules at low pH and negatively charged kaolinite surfaces. The pKa value of chitosan has been previously reported to be around 6.5–7.0. Most glucosamine units in chitosan are protonated at pH 3.5. Approximately half of the glucosamine units are protonated at pH 6.5, while most glucosamine units are not protonated at pH 8.5. These results show that increasing the acid concentration can increase the number of protonated glucosamine units, thereby enhancing the electrostatic interaction between kaolinite and chitosan. Moreover, under acidic conditions, the solubility of chitosan in water increases, facilitating the homogeneous adhesion of chitosan onto sand particles. Additionally, an optimal acid concentration is crucial for maximizing the dissolution of chitosan, ensuring that it evenly coats soil particles and improves composite integrity. However, excessively high acid concentrations can lead to over-protonation of chitosan, resulting in excessive positive charge that may repel soil particles rather than bind them effectively. This can decrease the overall stability and strength of the composite. Furthermore, very high acid concentrations can cause chitosan to precipitate out of the solution, reducing its effectiveness as a stabilizing agent. Therefore, maintaining an optimal acid concentration is key to achieving the best possible interaction and stabilization effect in chitosan-treated soils [47,48,49].

## 5. Comparison of Chitosan-Treated Soil Behavior with Other Biopolymer-Treated Soils

### 5.1. Biopolymer Content

**Figure 18:**
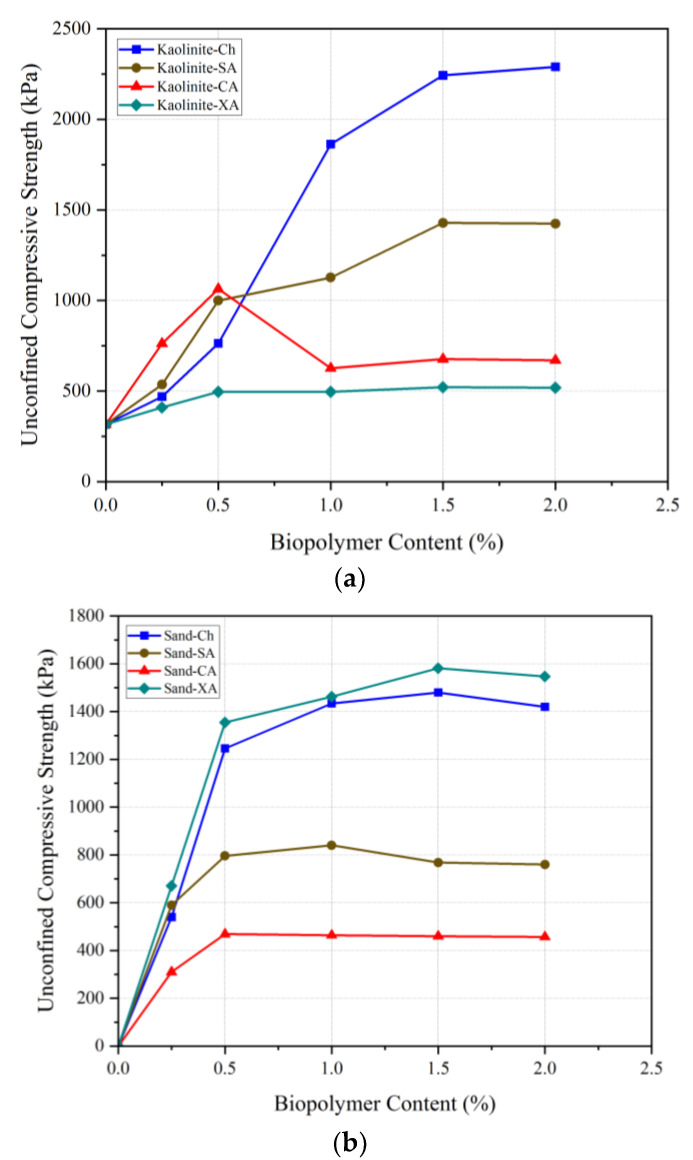
Different biopolymers’ effect on UCS improvement of (**a**) kaolinite and (**b**) sand.

This section presents chitosan’s performance in improving soil compression strength compared to some marine and conventional biopolymers, including sodium alginate (SA), carrageenan (CA), and xanthan (XA). As seen in Figure 18a, chitosan significantly outperforms other biopolymers in UCS enhancement of kaolinite. At 0.5% biopolymer content, chitosan outperformed xanthan, although sodium alginate and carrageenan exhibited better results. However, at biopolymer contents higher than 0.5%, chitosan significantly increased the strength, demonstrating the best performance compared to the other biopolymers. Particularly in terms of the comparison with xanthan, the most commonly used biopolymer in soil treatment, chitosan presented an exceptional performance, highlighting its potential as a great alternative to other common additives.

Chitosan also demonstrated remarkable effectiveness in stabilizing sandy soils. Its application led to a significant increase in UCS, from 0 kPa in untreated sand to 1480 kPa with only 1.5% chitosan. Even though xanthan achieved a UCS of 1581 kPa at 1.5% content, these results were not significantly higher than those achieved with chitosan. Also, carrageenan-treated sand reached a maximum UCS of 468.73 kPa at 0.5%, with minimal change at higher concentrations, while sodium alginate-treated sand peaked at 840 kPa with 1% content, showing a slight decline at higher dosages. The comparative results clearly establish chitosan as a highly effective biopolymer, outperforming others, including the commonly used xanthan, in kaolinite stabilization.

### 5.2. Long-Term Strength

**Figure 19:**
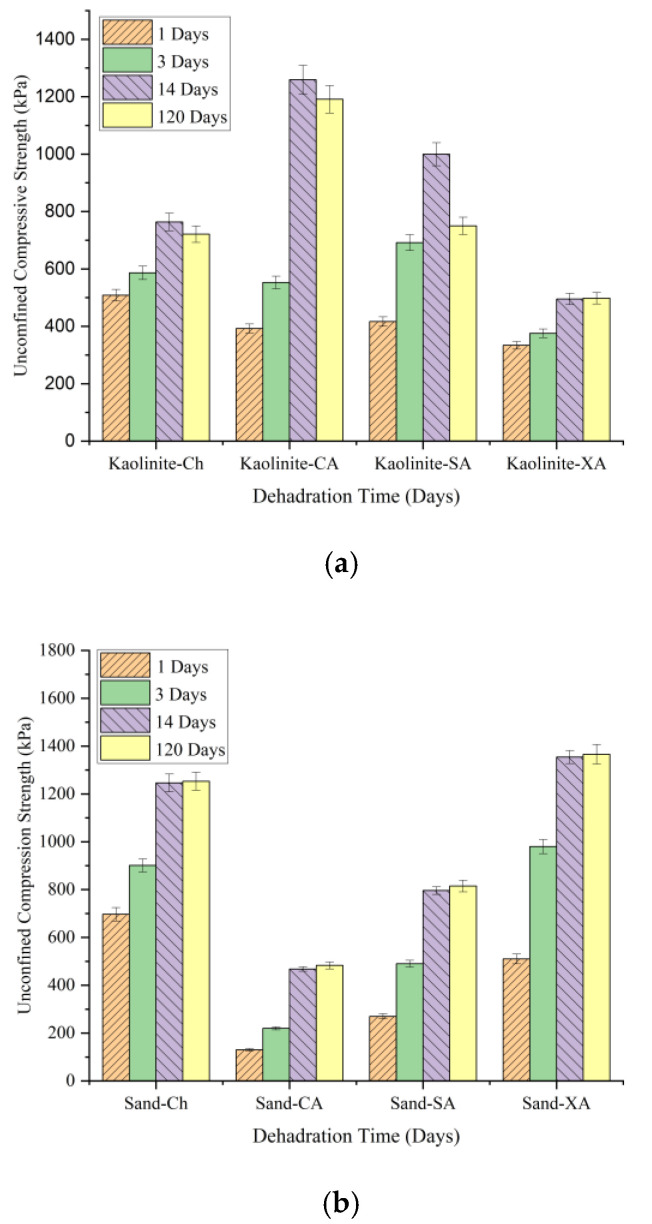
Long-term strength comparison of different biopolymers in stabilizing (**a**) kaolinite and (**b**) sand.

This section compares the long-term compressive strength of soils treated with chitosan (CH), carrageenan (CA), sodium alginate (SA), and xanthan gum (XA) over 1, 14, and 120 days of curing. Figure 19 illustrates the development of compressive strength for each biopolymer after 120 days. In kaolinite (Figure 19a), chitosan-treated samples provided a UCS increase from 508 kPa at 1 day to 763 kPa at 14 days, followed by a slight decrease to 721 kPa at 120 days. This suggests that while chitosan provides strong initial stabilization, it experiences minimal strength reduction over time. On the other hand, carrageenan-treated kaolinite peaked at a higher UCS of 1259 kPa at 14 days but decreased more significantly to 1191 kPa by 120 days. Sodium alginate- and xanthan-treated kaolinite showed smaller UCS, with sodium alginate decreasing from 1000 kPa to 750 kPa and xanthan stabilizing around 548 kPa. Chitosan-treated kaolinite retained nearly 95% of its strength after 120 days, indicating its strong potential for use in medium-term projects.

As shown in Figure 19b, all tested biopolymers maintained their strength effectively over a 120-day period. For sand, chitosan treatment improved the UCS from 697 kPa at 1 day to 1246 kPa at 14 days, with a slight increase to 1253 kPa at 120 days, indicating robust long-term strength. Although xanthan-treated sand outperformed chitosan with a UCS of 1366 kPa after 120 days, chitosan maintained its high strength. Sodium alginate and carrageenan gave relatively lower strengths. Overall, it can be concluded that chitosan provides a reliable performance and stability over time for both kaolinite and sand.

## 6. Conclusions

This study highlights the significant impact of chitosan biopolymer on the mechanical properties of sand and kaolinite soils. The key findings emphasize the effects of biopolymer content, long-term curing, temperature, and acid concentration on soil stabilization, particularly in enhancing unconfined compressive strength (UCS) and modulus of elasticity (EM).

The study demonstrates that chitosan significantly enhances the unconfined compressive strength (UCS) and elastic modulus (EM) of both sand and kaolinite soils. Sand achieved its maximum UCS at a chitosan content of 1.5%, reaching 1480 kPa, while kaolinite exhibited an optimal concentration of 2%, yielding a UCS of 2290 kPa. Although sand initially performed better due to its coarse-grained structure, facilitating effective mixing with chitosan, kaolinite surpassed it at higher biopolymer concentrations. This enhanced performance was attributed to stronger bonding mechanisms, including hydrogen bonding, electrostatic interactions, and hydrophobic bonding, which created a robust network that restricted soil particle movement and increased strength.

Long-term curing plays a critical role in the strength development of chitosan-treated soils, with both sand and kaolinite showing significant UCS increases over a 120-day period. The most notable strength gains occurred within the first seven days, particularly for sand, which exhibited a rapid increase in strength within one day due to its porous structure. UCS stabilization was observed after 14 days, highlighting the durability of chitosan-soil bonds over time. This strength enhancement was closely tied to the process of dehydration, where the reduction in moisture content reinforced the chitosan gel, allowing soils to achieve near-maximum strength within two weeks while maintaining structural integrity and low moisture levels over prolonged periods.

The efficacy of chitosan-stabilized soils is notably influenced by temperature. Experiments revealed that both sand and kaolinite experienced optimal performance at moderate curing temperatures around 45 °C, where optimal dehydration and stronger chemical bonding were observed. Beyond this range, excessive dehydration led to brittleness, with sand’s UCS declining above 45 °C and kaolinite achieving its peak strength at 60 °C before decreasing at 80 °C. Additionally, high temperatures, particularly at 80 °C, diminished the modulus of elasticity, most notably in sand, due to the formation of brittle chitosan fibers and a reduced adhesion surface area. These findings underscore the critical importance of maintaining moderate curing temperatures to enhance the strength and elasticity of chitosan-treated soils.

The dissolution of chitosan in acidic solutions plays a critical role in soil stabilization, as evidenced by UCS tests conducted with varying acetic acid concentrations (0% to 5%). The results indicate that a 0.5% acid concentration was most effective in enhancing chitosan solubility and improving soil strength. At lower concentrations (0–0.1%), chitosan dissolution was inadequate, leading to only slight strength improvements. In contrast, higher acid concentrations (5%) did not significantly affect UCS, highlighting an optimal range for chitosan solubility. Furthermore, the study underscores that the typical levels of acid rain encountered in the environment are far lower than those required to dissolve chitosan, suggesting that environmental acidification poses minimal risk to the stability of chitosan-treated soils.

The use of chitosan greatly enhances the shear strength characteristics of kaolinite. The cohesiveness of the material increased from 70 kPa to 192 kPa, while the internal friction angle increased from 23.76° to 34.58°. These enhancements may be attributed to the improved bonding between particles and the increased cohesion of the soil. The compaction process led to the creation of chitosan–soil conglomerates, which enhanced interlocking and frictional resistance. This highlights the efficacy of chitosan as a soil stabilizer.

## 7. Future Recommendations

In order to enhance the use of chitosan in soil stabilization, more investigation is required to optimize the chitosan concentration, duration of curing, and temperature for different soil compositions. Conducting extensive field trials is crucial to confirm the results obtained in the laboratory, while conducting thorough investigations on the environmental effect will guarantee the sustainable use of resources. In addition, conducting research on the use of different acids to dissolve chitosan and evaluating long-term durability would improve its practical use. Cost–benefit assessments are used to assess the economic viability of large-scale undertakings.

## Figures and Tables

**Table 1:** MH silt XRD analysis.

Component	Percentage (%)
SiO_2_	48.97
Al_2_O_3_	35.19
K_2_O	2.51
Fe_2_O_3_	0.88
MgO	0.35
TiO_2_	0.23

**Table 2:** The kaolinite silt’s geotechnical characteristics.

Soil Type	Sand Fraction (%)	Silt Fraction (%)	Clay Fraction (%)	L_L_ (%)	P_L_ (%)	P_I_ (%)	USCS	Activity = P_I_/Clay Content (%)
Kaolinite	0.88	78.00	21.12	62	46	16	MH	0.77

**Table 3:** XRD analysis of sand.

Component	Percentage (%)
Quartz	76.5
Calcite	3.4
Dolomite	0.1
Siderite	0.7
Siderite (Mg/Ca)	2.2
Andradite	1.0
Plagioclase (An0-15)	7.7
K-Feldspar	3.0
Kaolinite	0.9
Chlorite	1.4
Illite	3.2

**Table 5:** Properties of the chitosan biopolymer.

Product Name	Chitosan
Form	Solid
Chemical formula	{C6H11NO4}n
Appearance	Faint Beige to Beige
Melting point	>290 °C
Solubility in water	Insoluble in water and organic solvent, soluble in dilute aqueous acidic solution (pH < 6.5)
pH	6.5–7.5

**Table 4:** Geotechnical properties of the sand.

Soil Type	D_50_ (mm)	C_u_	C_c_	G_s_	Shape	UCSC	e_min_	e_max_
Sand	0.49	2.77	0.91	2.63	Round	SP	0.61	0.76

**Table 6:** Experimental program.

Test Type	Biopolymer Content (%)	Long Term Strength Assessment (Days)	Soil Type	Acid Concentration (g/L)	Temperature (°C)
UCS	0, 0.25, 0.5, 1, 1.5, 2	14	Kaolinite, Sand	0.5	25
	0, 0.5	0, 1, 3, 7, 14, 28, 60, 90, 120	Kaolinite, Sand	0.5	25
	0, 0.5	14	Kaolinite, Sand	0, 1, 2, 3, 4, 5, 10, 20, 50	25
	0, 0.5	14	Kaolinite, Sand	0.5	15, 25, 45, 60, 80
Triaxial	0, 0.5	14	Kaolinite, Sand	0.5	25
SEM	0, 0.5	14	Kaolinite, Sand	0.5	25

**Table 7:** Summary of the results of the CD triaxial test.

Sample ID	Biopolymer Content (%)	C’	φ’
Untreated kaolinite	0	70	23.73
Chitosan-treated kaolinite	0.5	192	34.58
Untreated sand	0	0	36
Chitosan-treated sand	0.5	161	39.5

## Data Availability

The original contributions presented in the study are included in the article; further inquiries can be directed to the corresponding authors.
